# Repeated determination of convective and radiative heat transfer coefficients using 32 zones thermal manikin

**DOI:** 10.1186/2046-7648-4-S1-A160

**Published:** 2015-09-14

**Authors:** Miloš Fojtlín, Jan Fišer, Miroslav Jícha

**Affiliations:** 1 Department of Thermodynamics and Environmental Engineering, Energy Institute, Faculty of mechanical engineering, Brno University of Technology, Czech Republic

## Introduction

Average European citizens spend 80 % to 90 % of their workday time indoors, in buildings or vehicles [1]. Owing to this fact, building design in terms of thermal comfort, air quality, and low energy demands is important. In addition, many independent studies provide evidence of improper thermal environment and the negative influence of this on the human body, e.g. [2], [3]. However, such situations can be tackled, in the future, using modern computational methods. To do so there is a need for anatomically detailed heat transfer coefficients that quantify heat flux from a human body. A lot of research has been done in order to investigate heat transfer coefficients in various body postures, wind speeds and wind directions, e.g. [4], [5], [6], [7]. On the other hand, there has not been any emphasis given to measurement reproducibility. A thermal manikin was involved to determine convective and radiative heat transfer coefficients in a sitting and standing posture repeatedly.

## Methods

Presented study involves the state-of-the-art thermal manikin (Newton). The manikin imitates human metabolic heat production, measures combined dry heat flux from its surface and also its surface temperature. To separate convective and radiative heat flux portion a low emissivity coating was applied to the surface of the manikin. Next, the manikin was placed into a calibration box that was built inside a climatic chamber to achieve uniform environment (t_amb _= t_rad _= 24 °C ± 0.2 °C, w = 0.05 m.s^-1^). Manikin's constant temperature control mode was set to 34 °C, as it is a reasonable approximation of a real human skin temperature in neutral thermal conditions. The measurement time schedule consisted of a period of the manikin preheating (1.5 to 3 h) and data logging (1 h). Each case was repeated three times.

## Results

Results of the research are presented via average values of convective and radiative heat transfer coefficients in a complete overview of 32 zones. Data of sitting and standing positions with 95% confidence error bars are available. The lowest values of convective heat transfer coefficients (h_c_) are found on the chest (3.3 W.m^-2^.K^-1^). The highest h_c _were indicated on the limbs (feet 6.6 W.m^-2^.K^-1^). Generally, h_c _values of the sitting manikin were slightly higher than those of the standing. Opposite logic applies in the case of radiative heat transfer coefficients (h_r_) where the (h_r_) maximum is on the back (5.5 W.m^-2^.K^-1^). Due to the manikin's hair, the lowest h_r _value was determined at the manikin's head (3 W.m^-2^.K^-1^).

## Discussion and conclusion

The results are compared with results of two independent authors [4], [5], [6]. Normally, differences among the authors in the mean coefficient values vary up to 1 W.m^-2^.K^-1 ^per segment. Extremes are found on the head, feet, seat, and back, where different conditions and geometry apply due to the different manikins involved. The trends of the results are mostly complying. In addition, thanks to the confidence bars we are also able to indicate systematic errors in measurements (lower limbs). To sum up, we have proven reproducibility of the method used, yet in a limited scale.

## Acknowledgements

The research was supported by the project LO1202 NETME CENTRE PLUS with the financial support from the Ministry of Education, Youth and Sports of the Czech Republic under the "National Sustainability Programme I". The authors gratefully acknowledge the support from the project No. FSI-S-14-2355 of the BUT.
